# Autochthonous Transmission of *Trypanosoma cruzi*, Louisiana

**DOI:** 10.3201/eid1304.061002

**Published:** 2007-04

**Authors:** Patricia L. Dorn, Leon Perniciaro, Michael J. Yabsley, Dawn M. Roellig, Gary Balsamo, James Diaz, Dawn Wesson

**Affiliations:** * Loyola University New Orleans, New Orleans, Louisiana, USA; † University of Georgia, Athens, Georgia, USA; ‡ Louisiana Office of Public Health, Metairie, Louisiana, USA; § Louisiana State University Health Sciences Center, New Orleans, Louisiana, USA; ¶ Tulane University Health Sciences Center, New Orleans, Louisiana, USA

**Keywords:** Chagas disease, United States, Louisiana, Triatoma, dispatch

## Abstract

Autochthonous transmission of the Chagas disease parasite, *Trypanosoma cruzi*, was detected in a patient in rural New Orleans, Louisiana. The patient had positive test results from 2 serologic tests and hemoculture. Fifty-six percent of 18 *Triatoma sanguisuga* collected from the house of the patient were positive for *T*. *cruzi* by PCR.

Chagas disease is endemic in Latin America; 13 million people are infected with the causative agent, the protozoan parasite *Trypanosoma cruzi*, and 200,000 new cases are reported annually (*1*). Although Chagas disease occurs mostly as heart disease, megasyndrome (enlargement of the visceral organs) is also seen in patients in South America. Transmission is usually by contamination of a person with parasite-laden feces of a triatomine bug (family *Reduviidae*, subfamily *Triatominae*, commonly known as kissing bugs), which deposits feces on the skin while feeding. The parasite can then enter through the bite wound, mucous membranes, or conjunctiva. Transmission by blood transfusion, organ transplant, and congenital and oral routes can also occur.

Only 5 autochthonous cases of infection with the Chagas disease parasite have been reported in the United States: 3 in infants in Texas (*2**,**3*), 1 in an infant in Tennessee (*4*), and 1 in a 56-year-old woman in California (*5*). The most important triatomine species in the United States for Chagas disease transmission are *Triatoma sanguisuga*, whose broad range extends across the southeast and reaches Maryland and Texas, and *T*. *gerstaekeri*, found in Texas and New Mexico (*6*). There is an active sylvan cycle in the United States; *T*. *cruzi* has been identified directly or by serologic analysis in ≥18 species of mammals (*7*), including raccoons, opossums, armadillos, foxes, skunks, dogs, wood rats, squirrels, and nonhuman primates (housed in outdoor research facilities). In Louisiana, *T*. *cruzi* infection has been identified in 28.8% and 1.1% of armadillos (*8**,**9*), 37.5% of opossums (*9*), 4.7% of rural dogs (*10*) and rarely in nonhuman primates (*11*, P.L. Dorn et al., unpub. data). The lack of human cases is usually attributed to not having a suitable habitat for the bugs in most US homes, a preference for animal hosts, and delayed defecation of triatomines found in the United States compared with those found in Latin America (*12*).

## The Study

In June 2006, a 74-year-old woman residing in a house in rural New Orleans was bothered by a considerable number (>50) of insect bites. The woman observed many bugs in the house and showed them to a fumigator, who identified them as triatomines. An internet search showed the potential for transmission of Chagas disease, and the woman sought help from a local health sciences center.

Serum samples from both residents of the house were tested for antibodies to *T*. *cruzi* at the Centers for Disease Control and Prevention (CDC) by an indirect fluorescent antibody (IFA) test. Samples were also tested at Loyola University (New Orleans, LA, USA) and then at CDC. by using an experimental dipstick assay (*Trypanosoma* Detect; InBios International Inc., Seattle, WA, USA). The woman resident was positive for antibodies to *T*. *cruzi* by IFA at dilutions of 1:128 (≈4 weeks after being bitten) and 1:64 (≈10 weeks after being bitten) and by dipstick assay. She was positive for trypanosomes by hemoculture testing with ≈10 mL blood and coculture in macrophages (*13*) ≈4 months after being bitten. Trypanosomes consistent with *T*. *cruzi* were observed in culture beginning on day 46 of culture, and amplification of a *T*. *cruzi*–specific 24Sα rRNA gene target confirmed that the isolate was *T*. *cruzi*. The other resident was negative by both serologic tests.

The index resident had a history of 5 trips to areas endemic for Chagas disease: Zacatecas, Mexico (1970); Cozumel, Mexico (1990); Belize (1991); Guatemala (19988); and Costa Rica (1998), each of <2 weeks duration, with stays in improved tourist hotels (less likely to harbor triatomines) except for the Belize trip, which included an ≈1-week stay in a palm thatch-roofed cabin. She had not used intravenous drugs or had a blood transfusion or organ transplant, and she is not the daughter of Latin American immigrants. Except for fatigue, the index patient had no symptoms and had an active lifestyle. Cardiac evaluation that included an electrocardiogram showed normal results, and she decided not to take medication.

Her residence of 29 years was located on 7.66 acres of bottomland hardwood forest, with many gaps that provided ready access for insects. A house inspection showed fecal streaks characteristic of triatomines on walls, which were identical to what the patient reported seeing on her nightgown. Twenty dead adult triatomines were collected in the house (after fumigation) and in another building on the property that contained a bed. No nymphs or eggs were found, which suggests that the house had not been colonized. One live second-stage nymph was collected in a nearby armadillo burrow ≈50 m from the house. All triatomines collected were identified as *T*. *sanguisuga* according to the key of Lent and Wygodzinsky (*6*) (Figure).

**Figure:**
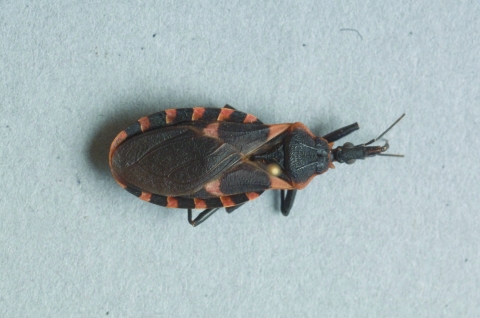
Male *Triatoma sanguisuga*.

Because all triatomines except the nymph were dead, PCR was used to determine *T*. *cruzi* infection status (*14*). The last 2 segments of the abdomen were removed from each insect, placed in 200 μL 1× PCR buffer (Applied Biosystems, Foster City, CA, USA), boiled for 15 min, and centrifuged. A total of 5 μL of supernatant was amplified in a 50-μL reaction (3.5 mmol/L MgCl_2_ and 2 U Taq DNA polymerase). The primers used anneal to the *T*. *cruzi* minicircle DNA and were TC3: 5′-TTGAACGCCCCTCCCAAAAC-3′ and TC4: 5′-GATTGGGGTTGGTGTAATATA-3′. The cycling parameters were an initial denaturation step at 94°C for 3 min; 35 cycles at 94°C, 55°C, and 72°C, each for 1 min; and a 10-min extension at 72°C (programmable thermal controller; MJ Research, Watertown, MA, USA). Twenty percent of the PCR product was subjected to electrophoresis on a 1.8% agarose gel and visualized by UV transillumination after staining with ethidium bromide. A positive control of 5 μL of *T*. *cruzi* parasites boiled in 1× PCR buffer and a negative control without the DNA template were included with every PCR. Samples that failed to amplify were spiked with 5 μL of *T*. *cruzi* parasites boiled in 1× PCR buffer and reamplified to ensure that the lack of product was not caused by inhibition of the PCR. More than half of the triatomines were positive for *T*. *cruzi* (56%, 10/18; 3 failed to amplify), with more positive females (73%, 8/11) than males (50%, 3/6). Plasma from the resident dog and 7 other dogs living ≈1 mile away all tested negative by IFA at CDC.

## Conclusions

The assertion that the patient contacted *T. cruzi* in Louisiana is strongly supported by limited travel history to disease-endemic areas and stays mostly in improved housing (risk for Chagas disease transmission is associated with longer residence in disease-endemic areas), lack of other risk factors, and large numbers of infected *T*. *sanguisuga* in the house. No periorbital swelling was reported. However, the streaks on her nightgown consistent with triatomine feces indicate exposure, and the parasite could have been introduced into any of her numerous bite wounds.

The residents had not previously noticed large numbers of *T*. *sanguisuga* in the house. However, Hurricane Katrina had hit the area 9 months earlier and increases in domestic infestation with triatomines have been previously reported after a hurricane (*15*). Anecdotally, the armadillo population increased substantially in the months after Hurricane Katrina, and one can speculate that these hosts supported a larger bug population, who later sought other bloodmeal sources as the armadillo population returned to prestorm levels. Follow-up studies of local *T*. *sanguisuga* ecology and animal reservoirs are planned.
